# Genetic Polymorphisms of Catalase and Glutathione Peroxidase-1 in Keratoconus

**Published:** 2018-10

**Authors:** Davood YARI, Ramin SARAVANI, Samira SARAVANI, Kamran EBRAHIMIAN, Hamid Reza GALAVI

**Affiliations:** 1. Cellular and Molecular Research Center, Zahedan University of Medical Sciences, Zahedan, Iran; 2. Dept. of Clinical Biochemistry, School of Medicine, Zahedan University of Medical Sciences, Zahedan, Iran; 3. Dept. of Biology, School of Fundamental Science, University of Zabol, Zabol, Iran; 4. Dept. of Molecular Biotechnology, Faculty of Pharmacy, University of Barcelona, Barcelona, Spain

**Keywords:** CAT, GPX-1, Oxidative stress, Keratoconus, Polymorphism

## Abstract

**Background:**

Keratoconus (KC) is a degenerative eye disease which results from thinning of the cornea and causes vision distortion. Oxidative stress damage to KC corneas may be because of the failure of corneas to process reactive oxygen species which leads to corneal thinning and loss of vision. Genetic variants in antioxidant defense genes such as catalase (CAT) and glutathione peroxidase (GPX) can decrease antioxidant capacity or increase oxidative stress and alter the risk of KC in patients. We investigated and evaluated the effects of single nucleotide polymorphisms in CAT, GPX-1 on the risk of KC in an Iranian population sample.

**Methods:**

This case-control study was performed on 140 patients with KC and 150 healthy control subjects in a sample of Iranian population from Zahedan, southern Iran in 2015. Genotyping of CAT rs7943316 and GPX-1 rs1050450 polymorphisms was done using polymerase chain reaction and restriction fragment length polymorphism (PCR-RFLP) method.

**Results:**

CAT rs7943316 A/T, AA genotype and A allele have a protective role against disease (OR =0.28, 95% CI =0.13–0.61, *P*=0.001 and OR = 0.50, 95% CI =0.35–0.72, *P*=0.0001, respectively) and decreased the risk of KC. Moreover, GPX-1 rs1050450 T allele increased the risk of KC in comparison with C allele (OR = 1.42, 95% CI = 1.01–2.03, *P*=0.03).

**Conclusion:**

CAT rs7943316 A/T, AA genotype, and A allele decreased the risk of KC. Moreover, in GPX-1 rs1050450 C/T polymorphism, T allele was associated with an increased risk of KC in our population.

## Introduction

Keratoconus (KC) is a degenerative eye disease which results from thinning of the cornea and causes vision distortion (1). This condition is characterized by the stromal thinning of the cornea which often results in bilateral and asymmetric corneal distortion and anterior corneal protrusion (2). Symptoms are highly variable and depend on the stage of the progression of the disorder. In the initial stage of the disease, there may be no symptoms; in the advanced stage, there is significant distortion of vision accompanied by serious visual loss. Patients with KC never become completely blind from their disease (3). KC is a major suggestion for corneal transplantation in developed countries(4). KC usually occurs in the second decade of life with progress in the next two decades and affects both genders and all ethnicities. The expected prevalence in the general population is 54 per 100000(5, 6). KC is most commonly an isolated disease, while several reports describe an association with Down syndrome, monosomy X (Turner syndrome), Leber’s congenital Ehlers-Danlos syndrome, neurocutaneousangiomatosis, neurofibromatosis, xerodermapigmentosa, collagenosis, retinitis pigmentosa and Marfan syndrome is described (7). The cause and original pathological mechanism are unknown but biochemical, genetic, and environmental factors are possible causes of KC and different etiologies may play a role in the development of this disease(8, 9).

Environmental factors which can influence on eye consist of rub eyeballs, allergic reaction, and solarization (9). An excess of environmental factors, particularly UV exposure, causes oxidative damage to KC corneas because of the failure of KC corneas to process reactive oxygen species (ROS) which leads to corneal thinning and loss of vision (10). The accumulation of ROS can damage cells by reacting with proteins, DNA, and membrane phospholipids (11). The normal cornea’s antioxidant enzymes eliminate the ROS before they damage cells; these consist of superoxide dismutase, glutathione reductase, catalase, and glutathione peroxidase, but in the disease condition, ROS can devastate cellular defenses and promote cell damage (12).

Catalase (CAT) is a common enzyme that found in the peroxisome of many aerobic organisms. This enzyme is a tetramer with molecular weight of about 240000 with four heme groups per tetramer (13, 14). Hydrogen peroxide (H_2_O_2_) is converted to H_2_O and O_2_ by CAT and thereby mitigates the toxic effects of hydrogen peroxide (15). Location of CAT gene is on chromosome 11p13 and comprise 13 exons; rs7943316 (-21 A/T) settled in nearly to the start site at promoter region (16). Deficiency of catalase may cause elevated concentrations of hydrogen peroxide and increase the risk of the progress of pathologies (17). Glutathione Peroxidase-1(GPX-1) encodes a member of the glutathione peroxidase family. GPX-1 is an intracellular antioxidant enzyme that converts H_2_O_2_ to water and protects the organism from oxidative damage(18, 19). GPx-1 is selenium-containing Enzyme, located at chromosome 3p21.3(20). GPx-1 has four SNPs that alter the amino acid produced but only one has been studied extensively in human disease, including rs1050450 (or GPx1 Pro197Leu). This C>T variation changes the amino acid from proline (Pro) to leucine (Leu) at position 197 (21). The GPx-1 variant may be associated with a decreased ability to scavenge ROS (22).

To the best of our knowledge, no studies have investigated the role of antioxidant gene CAT and GPX polymorphisms for KC patients. The present study aimed to evaluate the impact of CAT rs7943316 A/T and GPX-1 rs1050450 C/T polymorphisms on KC patients in a sample of Iranian population. These polymorphisms may alter the enzymes’ antioxidant capacity and may be associated with the risk of KC induced by oxidative damage.

## Materials and Methods

### Patients

This case-control study was done on 140 patients with KC (61 men and 79 women), and 150 healthy individuals as the control group (65 men and 85 women), who were enrolled from Alzahra Eye Hospital, Zahedan, southern Iran in 2015.

Ethical approvals for recruitment were obtained from the local Ethics Committee of Zahedan University of Medical Sciences and informed consents were obtained from all patients and healthy individuals. Detection of KC was done under criteria previously mentioned (23).

### Sampling and extraction of DNA

Venus blood samples were collected from the patients and healthy controls, using EDTA-containing tubes, and DNA was extracted using the salting-out method (24). The quality of the isolated DNA was verified using electrophoresis on 1% agarose gel and quantitated spectrophotometrically and stored, at -20°C until further use.

### Genotyping of CAT rs7943316 A/T Polymorphism

**Fig. 1:**
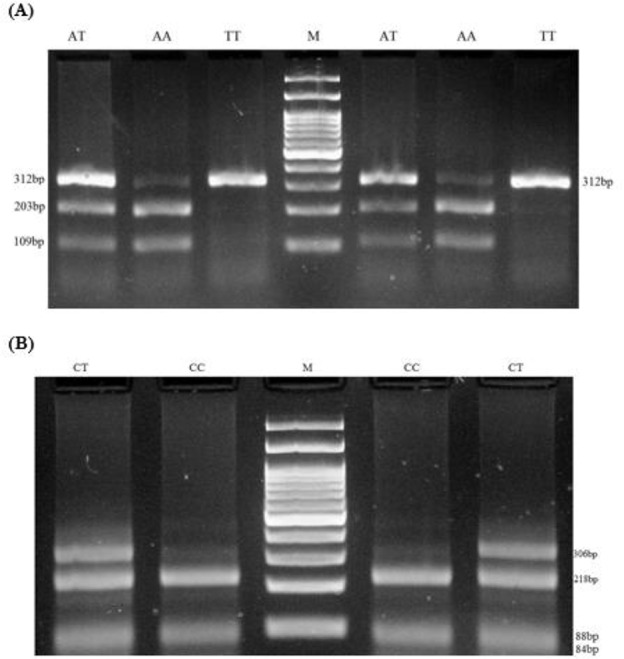
Electrophoresis pattern for detection of SNP in CAT rs7943316 A/T and GPX-1 rs1050450 C/T **(A)** RFLP -PCR products of CAT rs7943316 A/T, M: DNA marker (100 bp), product size (109, 203, 312bp), demonstrate genotype AT; product size(109, 203bp), demonstrate genotype AA and 312bp demonstrate genotype TT. **(B)** RFLP -PCR products of GPX-1 rs1050450 C/T, M: DNA marker (100 bp), product size (84/88, 218, 306bp), demonstrate genotype CT; product size(84/88, 218bp), demonstrate genotype CC

The restriction fragment length polymorphism, polymerase chain reaction (RFLP -PCR), was used for genotyping CAT rs7943316 polymorphism using forward and reverse primer sequences as follows: 5′-CTTCCAATCTTGGCCT-GCCTAG -3′ and 5′-CCGCTTTCTAAACGGACCTTCG-3′, respectively(25). PCR was directed using PCR master mix (AmpliqonTaq 2x mastermix, Denmark) according to the manufacturer’s instructions. The following conditions were involved for amplification of SNPs in (20 μL volume): 1 μL template DNA (∼100 ng/ μL), 1 μL of each primer (10 pmol/μL), 10 μL mastermix and 7 μLDNase free water were added. PCR product size was 312 bp. The PCR cycling conditions consisted of an initial denaturing step for 5min at 95°C followed by 30 cycles for 30 sec at 95°C, 40 sec at 60°C, and 30 sec at 72°C, anda final extension step for 5 min at 72°C. The PCR products were visualized on a 2% agarose gel containing 0.5 *μ*g/mL of ethidium bromide. Amplified DNA was digested with restriction enzyme HinfI (BIORON, Germany) at 37 °C for 3 h according to the manufacturer’s instructions. HinfI digestion resulted in two 109bp and 203bp fragments for AA genotype, three fragments of 312, 203 and 109bp for AT genotype, and no digested product is a 312bp for TT genotype(Fig. 1A).

### Genotyping of GPX-1 rs1050450 C/T Polymorphism

Genotyping of GPX-1 rs1050450 was done by RFLP-PCR method. Briefly, forward and reverse primers were 5′-TTATGACCGACCCCAAGCTC-3′ and 5′-GACACCCGGCACTTTATTAGTG -3, respectively (25). The PCR conditions were justified as follows: 5 min preheating at 95 °C, 30 cycles of 95 °C for 30 sec, 61 °C for 25 sec, and 72 °C for 28 sec followed by a final extension step for 5 min at 72 °C. PCR product size was 312 bp. Ten microliters of PCR product were later digested by ApaI restriction enzyme (Thermo Scientific, Lithuania) at 37 °C for 3 h. C allele produced 84, 88 and 218bp patterns, while T allele produced 84 and 306bp (Fig. 1B).

### Statistical analysis

The statistical analysis was performed using SPSS ver. 19.0 software (Chicago IL, USA). Comparison of quantitative variants between two groups was assessed by Student’s *t*-test. The frequencies of the alleles and genotypes were analyzed using the χ^2^-test or Fisher’s exact test. The odds ratio(OR) and 95% confidence intervals (CI) were also estimated. *P*-value < 0.05 was considered statistically significant.

## Results

No significant differences were observed between the patients with KC and healthy individual concerning gender and age (patients: 140, aged 10 to 80 yr and mean ± SD = 28 ± 12.5, *P*-value: 0.2 and controls: 150, aged 8 to 83 yr and mean ± SD = 29.9 ± 15.6, *P*-value: 0.2).

Table 1, containing the genotypes and allele distribution of CAT rs7943316 and GPX-1 rs1050450 variants in KC patients and control group. In fact, Table 1 shows the relationship between gene variants (SNP) and number of patients with KC and healthy controls was tested by computing the odds ratio (OR) and 95% confidence intervals (95% CI) from logistic regression analyses.

**Table 1:** The genotypes and allele distribution of CAT and GPX-1 variants in keratoconus (KC) patients and control group

***Variants***	***KC Patients n (%)***	***Controls n (%)***	***OR(95 % CI)***	***P-Value***
rs7943316, CAT				
TT	88 (62.9)	71 (47.3)	Ref.	—
TA	41 (29.2)	48 (32)	0.69 (0.41–1.16)	0.161
AA	11 (7.9)	31 (20.7)	0.28 (0.13–0.61)	0.001
Allele				
T	217 (77.5)	190 (63.3)	Ref.	—
A	63 (22.5)	110 (36.7)	0.50 (0.35–0.72)	0.0001
rs1050450, GPX-1				
CC	40 (28.6)	66 (44)	Ref.	—
CT	100 (71.4)	84 (56)	1.96 (1.21–3.20)	0.007
TT	0 (0)	0 (0)	—	—
Allele				
C	180 (64.3)	216 (72)	Ref.	—
T	100 (35.7)	84 (28)	1.42 (1.01–2.03)	0.03

The frequency distributions of CAT rs7943316 A/T genotypes in KC patients were: TT, 62.9%, TA, 29.2% and AA, 7.9%; and the distribution in healthy controls were: TT, 47.3%, TA, 32%, and AA, 20.7%. This SNP was associated with KC. The AA and A allele decreased the risk of KC (OR = 0.28, 95% CI = 0.13–0.61, *P*-value=0.001 and OR =0.50, 95% CI =0.35–0.72, *P*-value =0.0001, respectively) (Table 1).

The frequencies distributions of CC and CT genotypes of GPX-1 rs1050450 C/T gene polymorphism in KC patients were: 28.6% and 71.4% and in healthy controls were: 44% and56%, respectively. The T allele was associated with KC. Thers1050450 T allele increased the risk of KC in comparison with C allele (OR =1.42, 95% CI =1.01–2.03, *P*-value= 0.03) (Table 1).

The possible association between polymorphisms and clinical and pathological characteristics were analyzed. Significant association was observed between GPX-1 rs1050450 polymorphism with level of KC and cross-linking (CXL) (*P*-value= 0.04 and 0.04, respectively) (Table 2).

**Table 2:** Association between the CAT rs7943316 and GPX-1 rs1050450 polymorphisms and clinicopathological characteristics

***Parameters evaluated***	***Patients n (%)***	***(rs7943316) P-value***	***(rs1050450) P-value***
KC ocular			
OD	42(30.0)		
OS	36(25.7)	0.95	0.05
OU	62(44.3)		
Level of KC			
KK 1	33(23.6)		
KK 2	45(32.1)	0.82	0.04
KK 3	62(44.3)		
CXL			
OD	39(27.9)		
OS	40(28.6)	0.45	0.04
OU	42(30.0)		
Candidate	19(13.6)		

KC: keratoconus, OD (right eye), OS (left eye), OU (both eyes), CXL (cross linking surgery)

We analyzed the possible association between polymorphisms and clinical and pathological characteristics (Table 2). KC ocular showed the existence of keratoconus in a patient’s eye and relation with SNPs. Level of KC represents the level of disease and evaluating these criteria with polymorphisms from patients and our findings were not associated with KC. CXL represents number of persons has cross-linking surgery and relation with SNPS.

## Discussion

We investigated the impact of CAT rs7943316 A/T and GPX-1 rs1050450C/T polymorphisms on KC risk in a sample of Iranian population because these polymorphisms may alter the enzymes’ antioxidant capacity, leading to synergistic effects with KC induced by oxidative damage.

Our results indicated that CAT rs7943316 A/T, AA genotype, and A allele significantly decreased the risk of KC. Moreover, GPX-1 rs1050450 C/T, T allele was associated with an increased risk of KC in our population. Genetic variations in antioxidant defense may decrease antioxidant capacity or increase oxidative stress and alter the risk of KC in patients. SNPs and gene variants suggest an intricate aetiology or convergence of multiple disease pathways. Elevation of oxidative stress from SNPs in specific antioxidant enzymes maybe associated with disease (21).

Inflammation plays a crucial role in the pathogenesis of KC. Corneal thinning and distortion in KC associated with some features of inflammation in ocular rosacea (26). Further evidence emphasizes the reduced levels of antioxidant defense enzymes in KC to remove ROS associated with inflammatory reactions (27). Oxidative stress results from increased ROS or decreased levels of antioxidants (28). Ultraviolet light (UV) is a source of ROS, and excessive exposure to sunlight leads to oxidative damage to KC corneas, where there is a reduced amount of the enzymes including CAT and GPX-1 necessary to remove the ROS (29). UV-B is damaging to the cornea, and UV-A damages both the cornea and lens (30). The effects of sunlight on the eye may be acute after a latent period of several hours. UV-B can foundation homolytic fission of H2O2 to generate hydroxyl radical (31). Moreover, there is an accumulation of oxidized mitochondrial DNA in KC relative to control corneas (32). The normal cornea’s antioxidant enzymes such as CAT and GPX eliminate the ROS before they damage cells, but in the disease condition, ROS can devastate cellular defenses and promote cell damage (12). KC corneas do not eliminate ROS in a normal way, which may play a major role in the pathogenesis of this disease(33).

Irregular expression of several major antioxidant enzymes including glutathione activities has been reported in KC corneas (34). In KC corneas compared with normal corneas, striking decreased in antioxidant potency and glutathione content (35). The KC corneas were exhibited a 2.2-fold increase of catalase mRNA level and 1.8-fold of enzyme activity. Since H2O2 induces catalase expression, KC corneas had high levels of this ROS, explaining some oxidative damages related with KC corneas (36). The elevated levels of oxidative stress products and decreased anti-oxidant capacity and antioxidant defenses in KC corneas indicate that oxidative stress may be involved in the progress of this pathology (35).

CAT rs7943316 and GPX-1 rs1050450 polymorphisms alter their antioxidant capacity and associated with different kinds of diseases such as Kashin-Beck (37), prostate cancer (38), peripheral neuropathy (39), diabetes mellitus (16) recurrent miscarriage (40), and brain tumors (41). In opposite to our findings, CAT rs7943316 genotype (TT) was associated with an increased risk of T2DM and no evidence was found to support an association between GPx-1 (198 C/T) polymorphism and patients with T2DM (25). Allele and genotype frequencies of the CAT–21A/T and GPX1–198C/T, variations on patients with cataract and significant differences were not observed compare controls (41); both in terms of disease and result obtained did not establish relationship with our results.

The variability of the XRCC1, POLG and FEN1 genes in the base excision repair pathway may play a role in KC pathogenesis and increase the risk of this disease (42, 43). Patients with KC corneas that have lower paraoxonase activity there were sensitive to oxidative stress (44).

The current report is the first to suggest an association between the polymorphisms of antioxidant enzyme genes with KC in the Iranian population. However, the present study has some limitations. For example, we examined just one polymorphism from each gene associated with KC and we did not consider different ethnics.

## Conclusion

CAT rs7943316 decreased the risk of KC. Moreover, GPX-1 rs1050450 T allele increased the risk of KC in the study population. Further studies with different ethnicities and nationalities are required to confirm our findings.

## Ethical considerations

Ethical issues (Including plagiarism, informed consent, misconduct, data fabrication and/or falsification, double publication and/or submission, redundancy, etc.) have been completely observed by the authors.
